# Supplementary figures and images for: Targeting DNA topoisomerases or checkpoint kinases results in an overload of chaperone systems, triggering aggregation of a metastable subproteome
Source: eLife. 2022 Feb 24;11:e70726. doi: 10.7554/eLife.70726 (PMC8871389; doi:10.7554/eLife.70726)

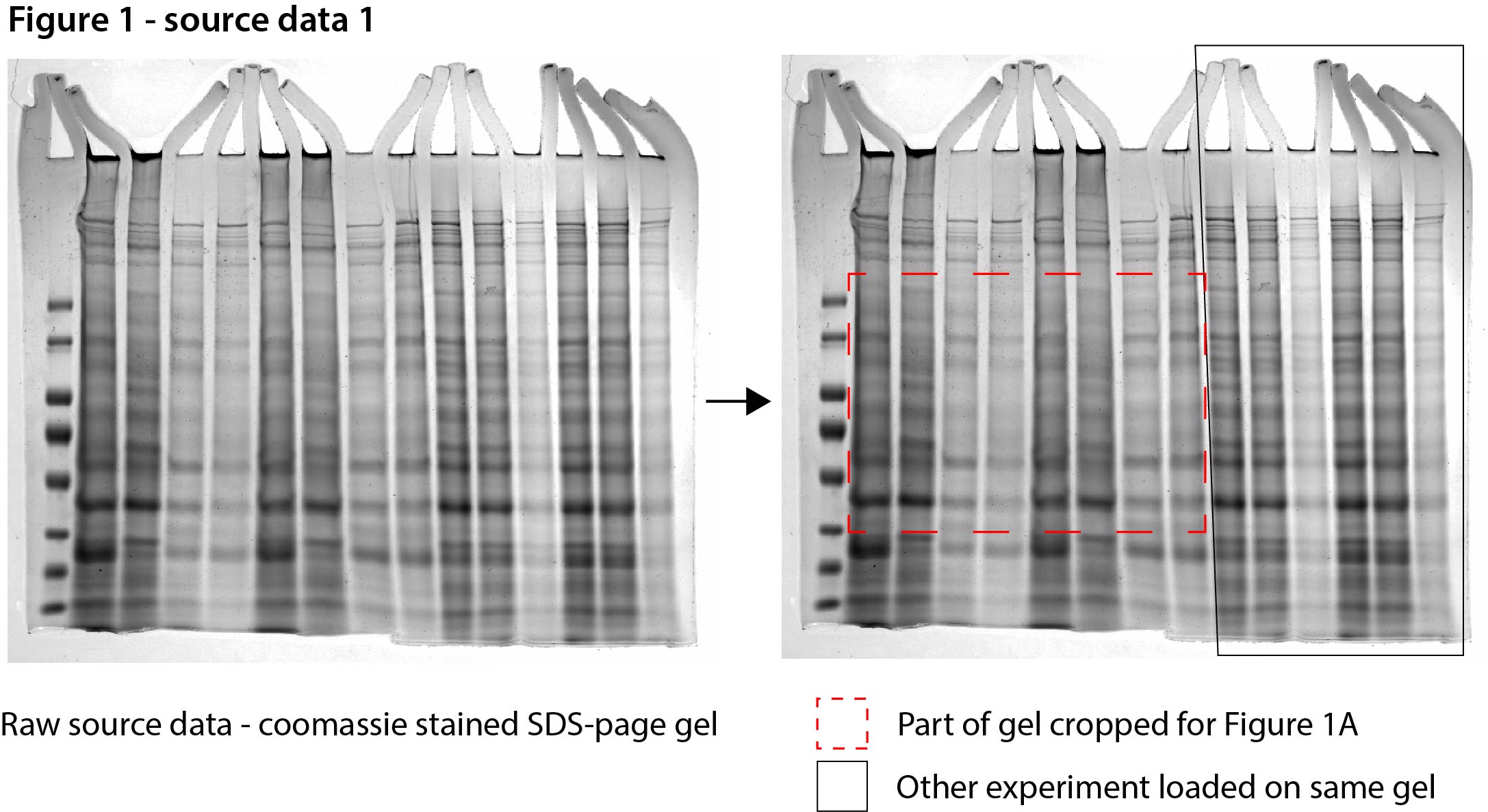

Supplement: Figure 1—source data 1. — Raw Coomassie-stained SDS-PAGE gel with cropped parts indicated in red. [file elife-70726-fig1-data1.jpg]

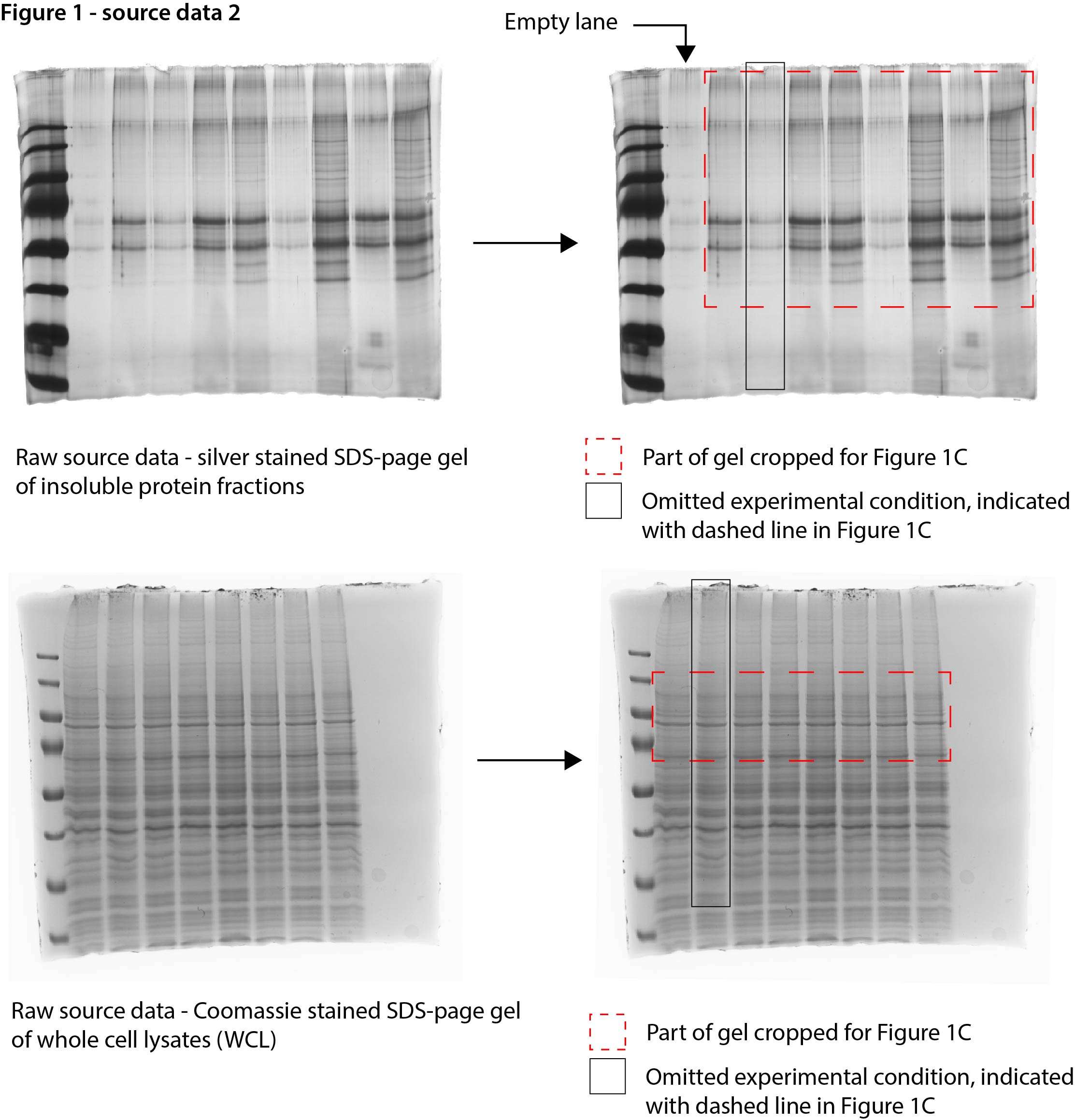

Supplement: Figure 1—source data 2. — Raw Coomassie- and silver-stained SDS-PAGE gels with cropped parts indicated in red. [file elife-70726-fig1-data2.jpg]

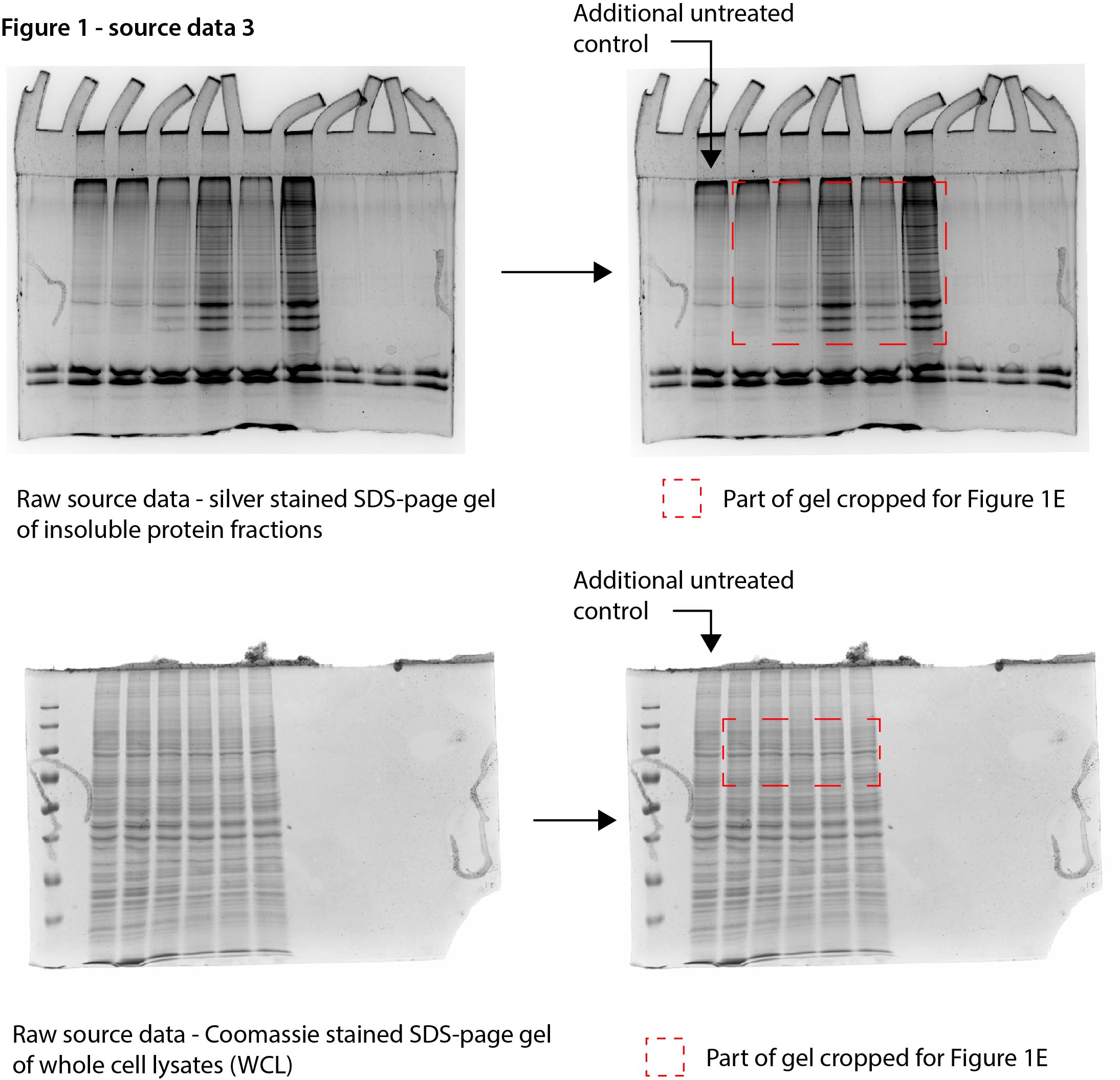

Supplement: Figure 1—source data 3. — Raw Coomassie- and silver-stained SDS-PAGE gels with cropped parts indicated in red. [file elife-70726-fig1-data3.jpg]

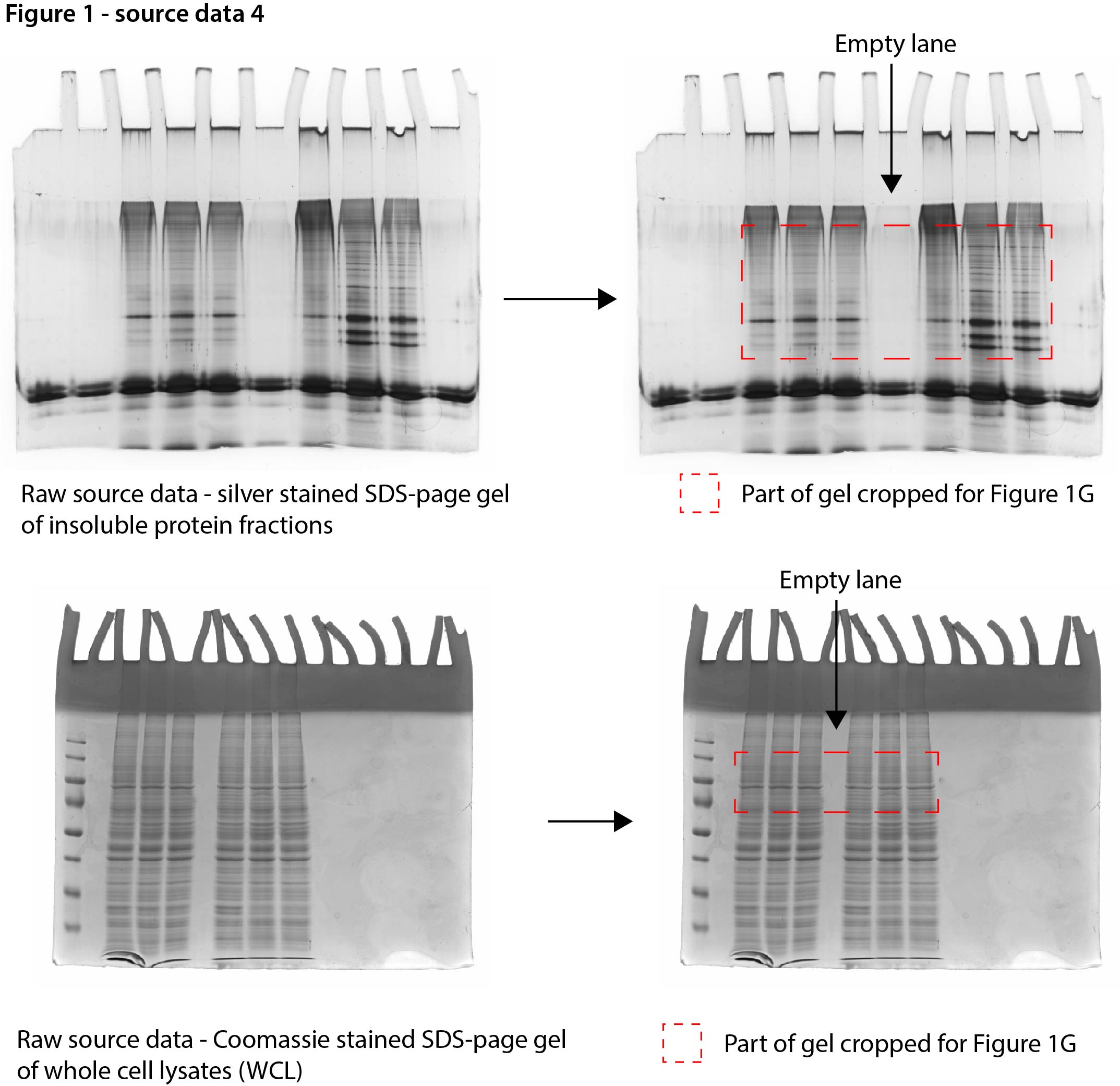

Supplement: Figure 1—source data 4. — Raw Coomassie- and silver-stained SDS-PAGE gels with cropped parts indicated in red. [file elife-70726-fig1-data4.jpg]

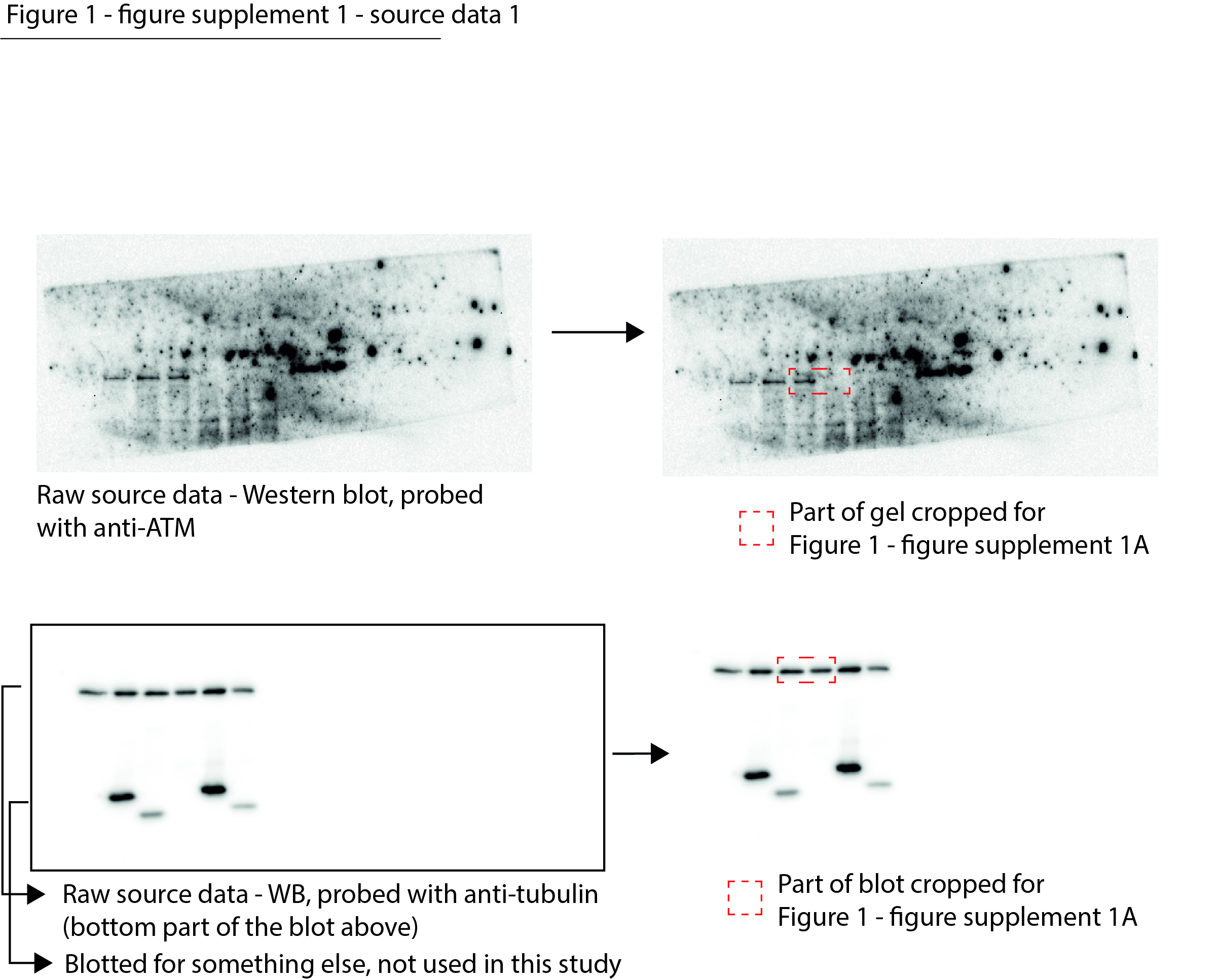

Supplement: Figure 1—figure supplement 1—source data 1. — Raw Western blot images with antibodies annotated and cropped parts indicated in red. [file elife-70726-fig1-figsupp1-data1.jpg]

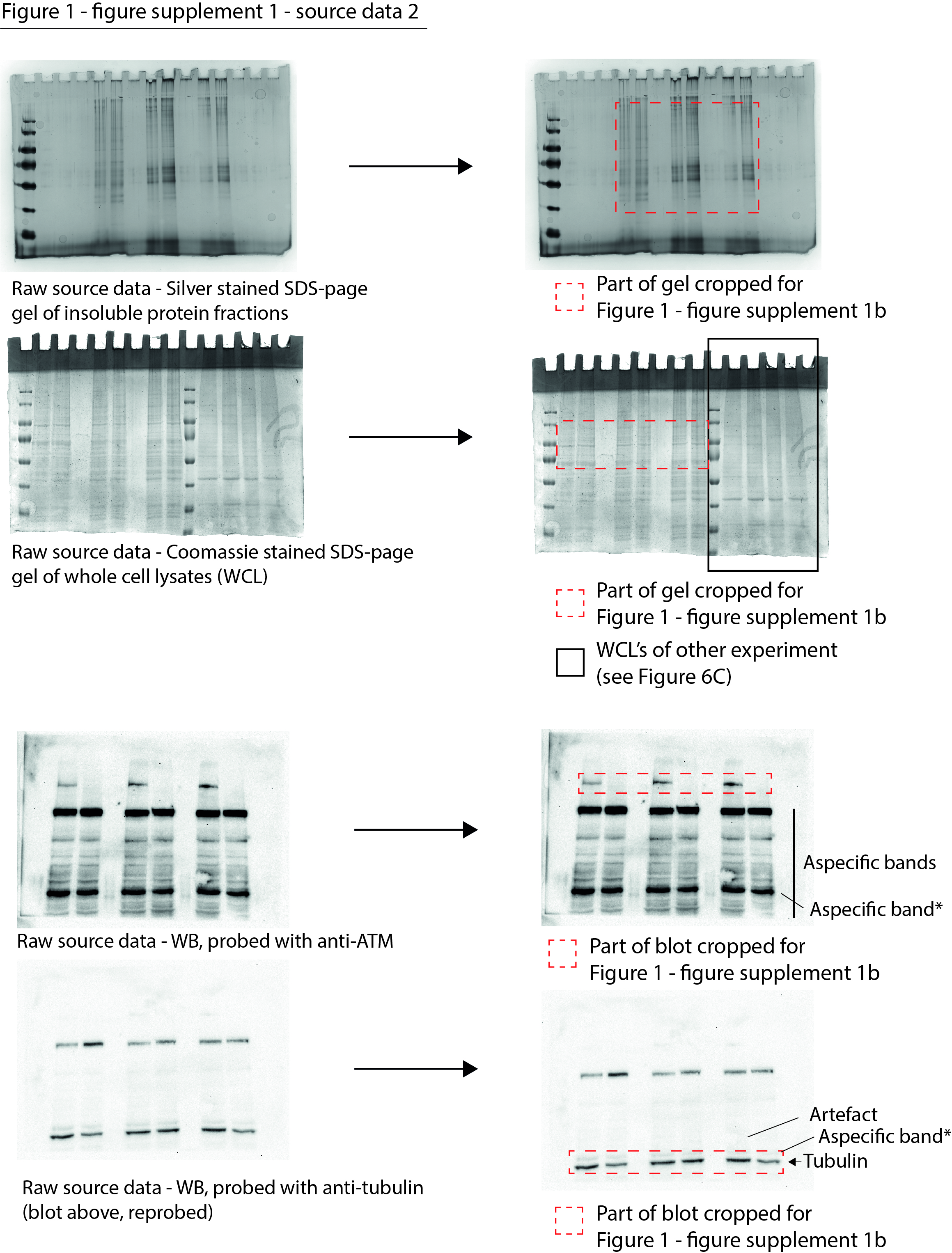

Supplement: Figure 1—figure supplement 1—source data 2. — Raw Coomassie- and silver-stained SDS-PAGE gels and Western blot images with antibodies annotated and cropped parts indicated in red. [file elife-70726-fig1-figsupp1-data2.jpg]

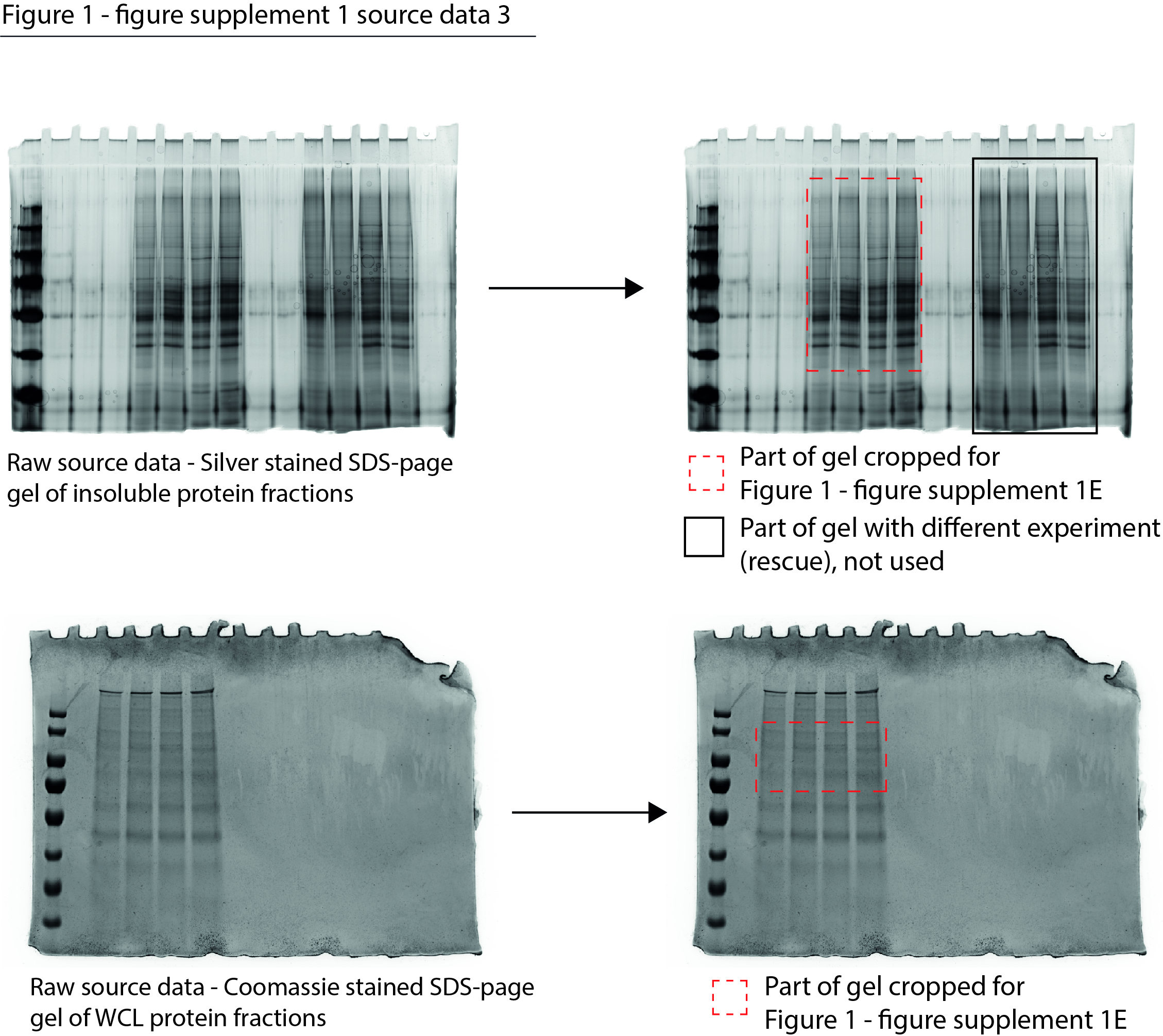

Supplement: Figure 1—figure supplement 1—source data 3. — Raw Coomassie- and silver-stained SDS-PAGE gels with cropped parts indicated in red. [file elife-70726-fig1-figsupp1-data3.jpg]

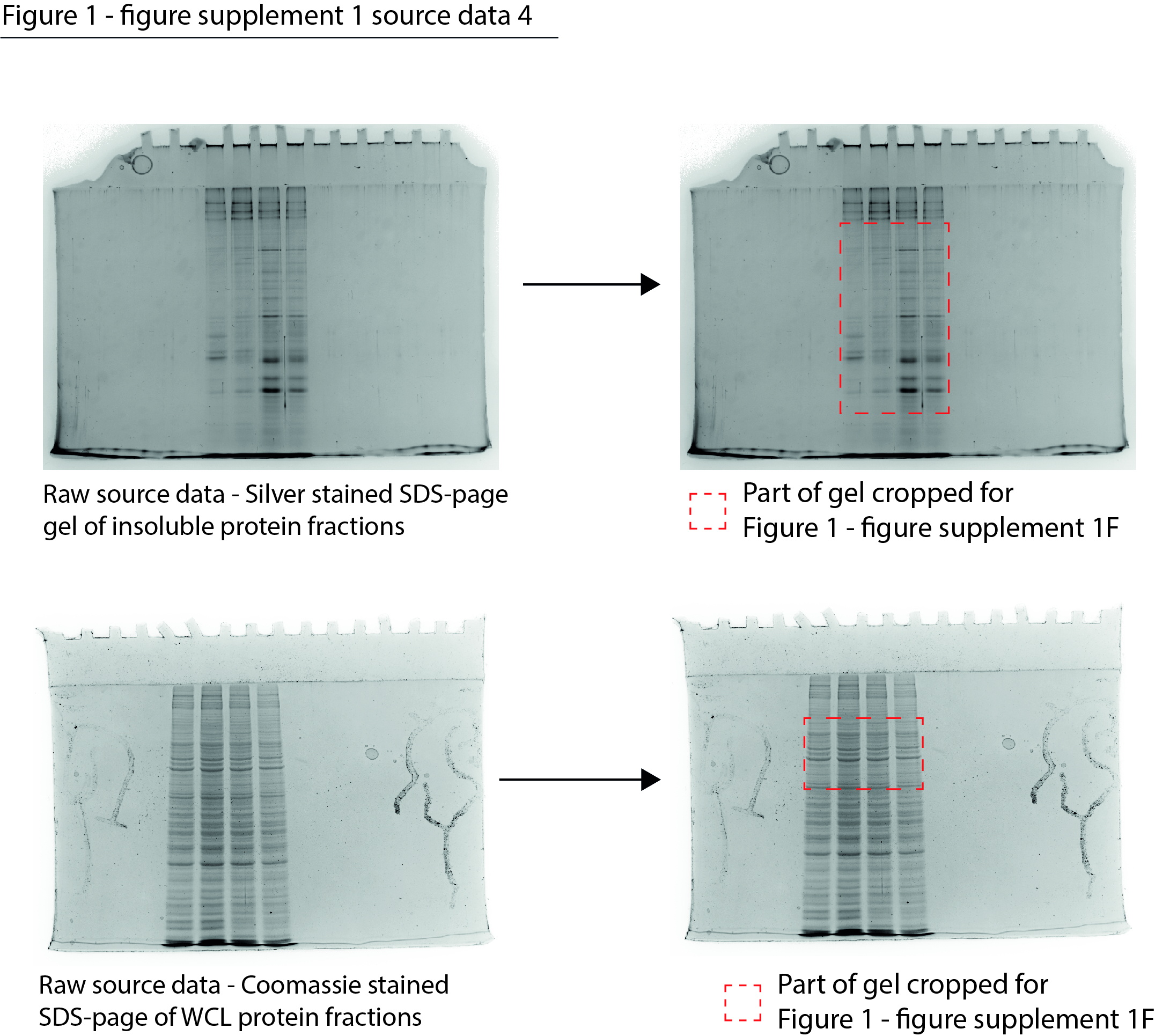

Supplement: Figure 1—figure supplement 1—source data 4. — Raw Coomassie- and silver-stained SDS-PAGE gels with cropped parts indicated in red. [file elife-70726-fig1-figsupp1-data4.jpg]

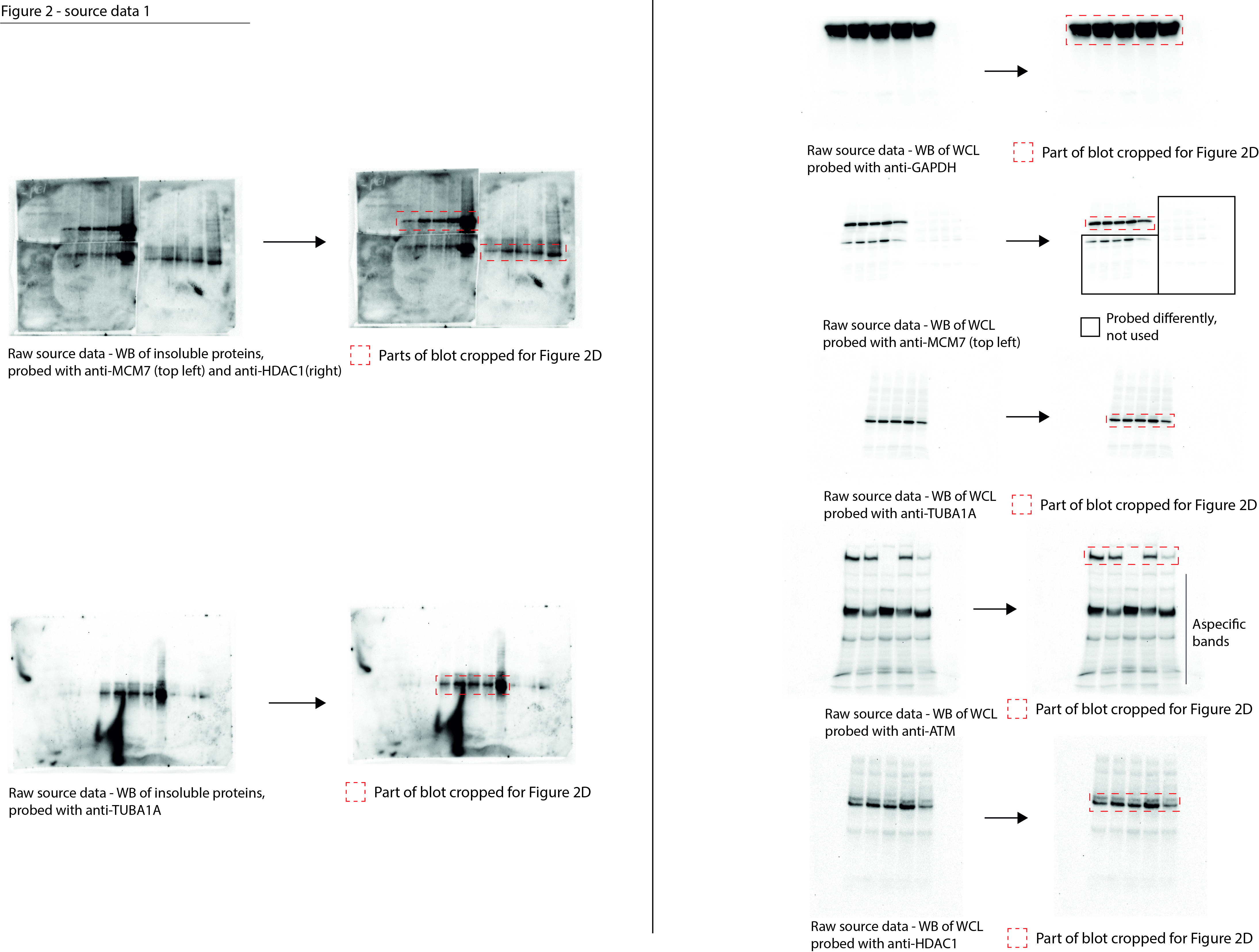

Supplement: Figure 2—source data 1. — Raw Western blot images with each antibody annotated and cropped parts indicated in red. [file elife-70726-fig2-data1.jpg]

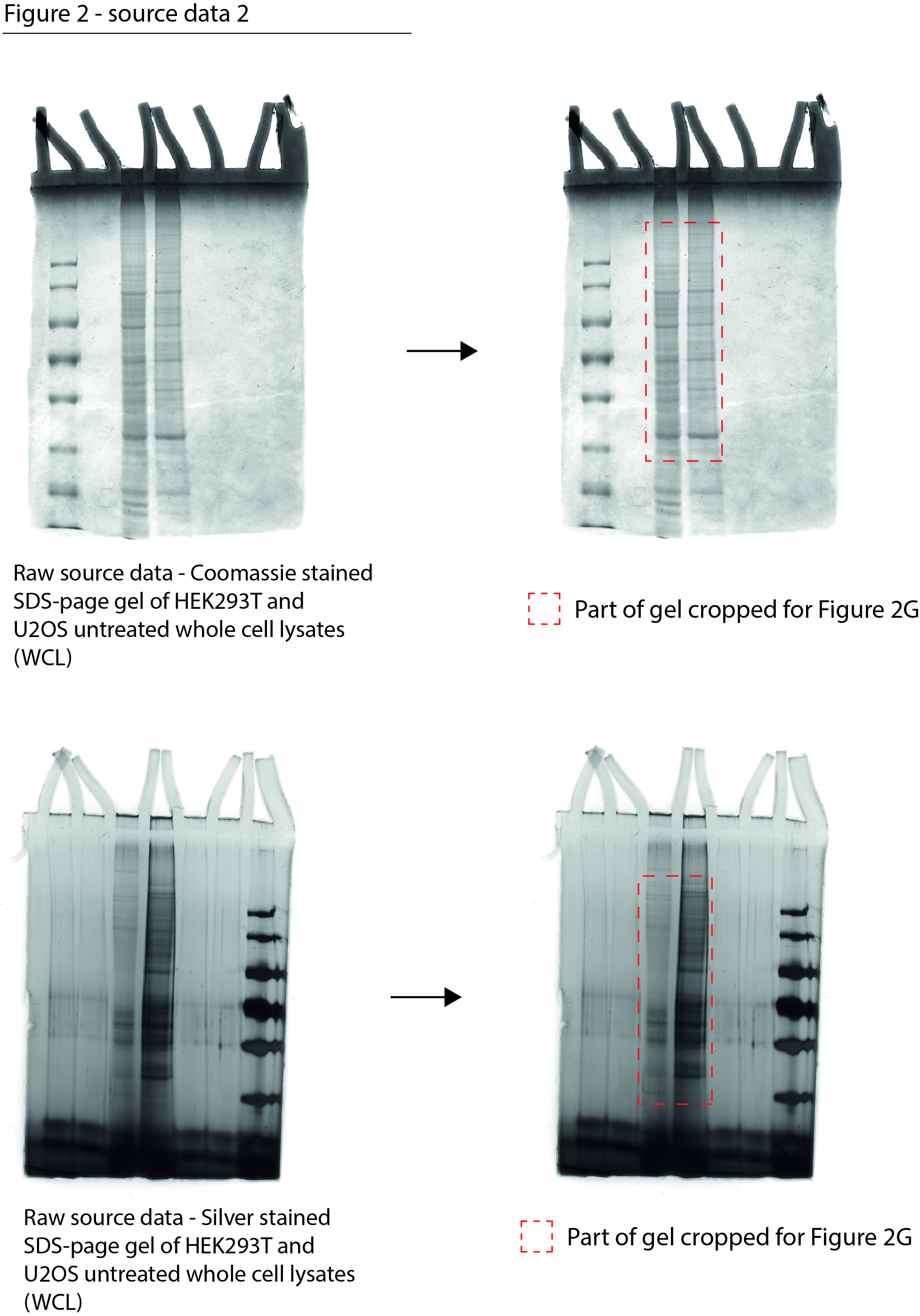

Supplement: Figure 2—source data 2. — Raw Coomassie- and silver-stained SDS-PAGE gels with cropped parts indicated in red. [file elife-70726-fig2-data2.jpg]

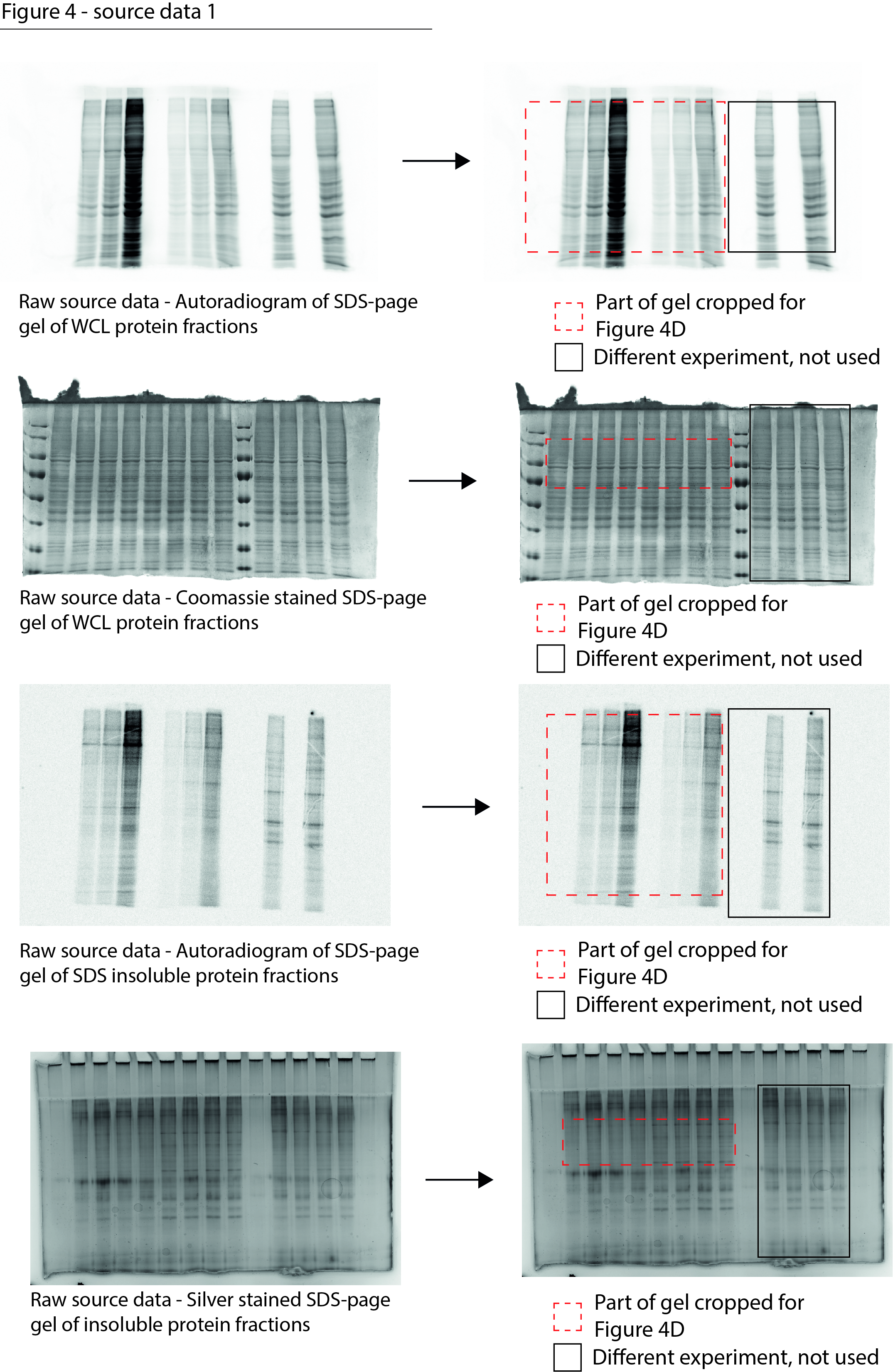

Supplement: Figure 4—source data 1. — Raw autoradiograms and Coomassie- and silver-stained SDS-PAGE gels with cropped parts indicated in red. [file elife-70726-fig4-data1.jpg]

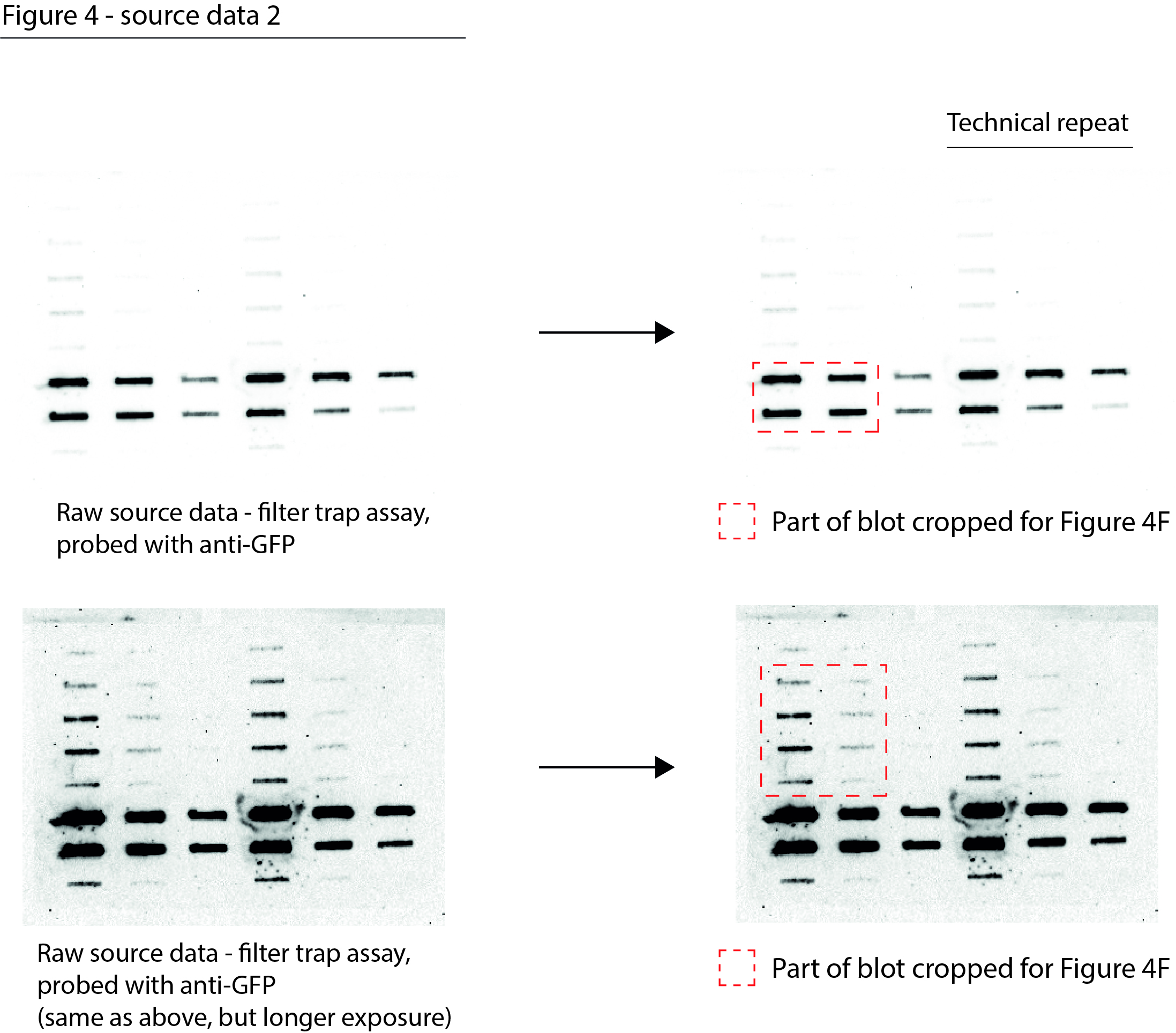

Supplement: Figure 4—source data 2. — Raw filter trap images with the cropped parts indicated in red. [file elife-70726-fig4-data2.jpg]

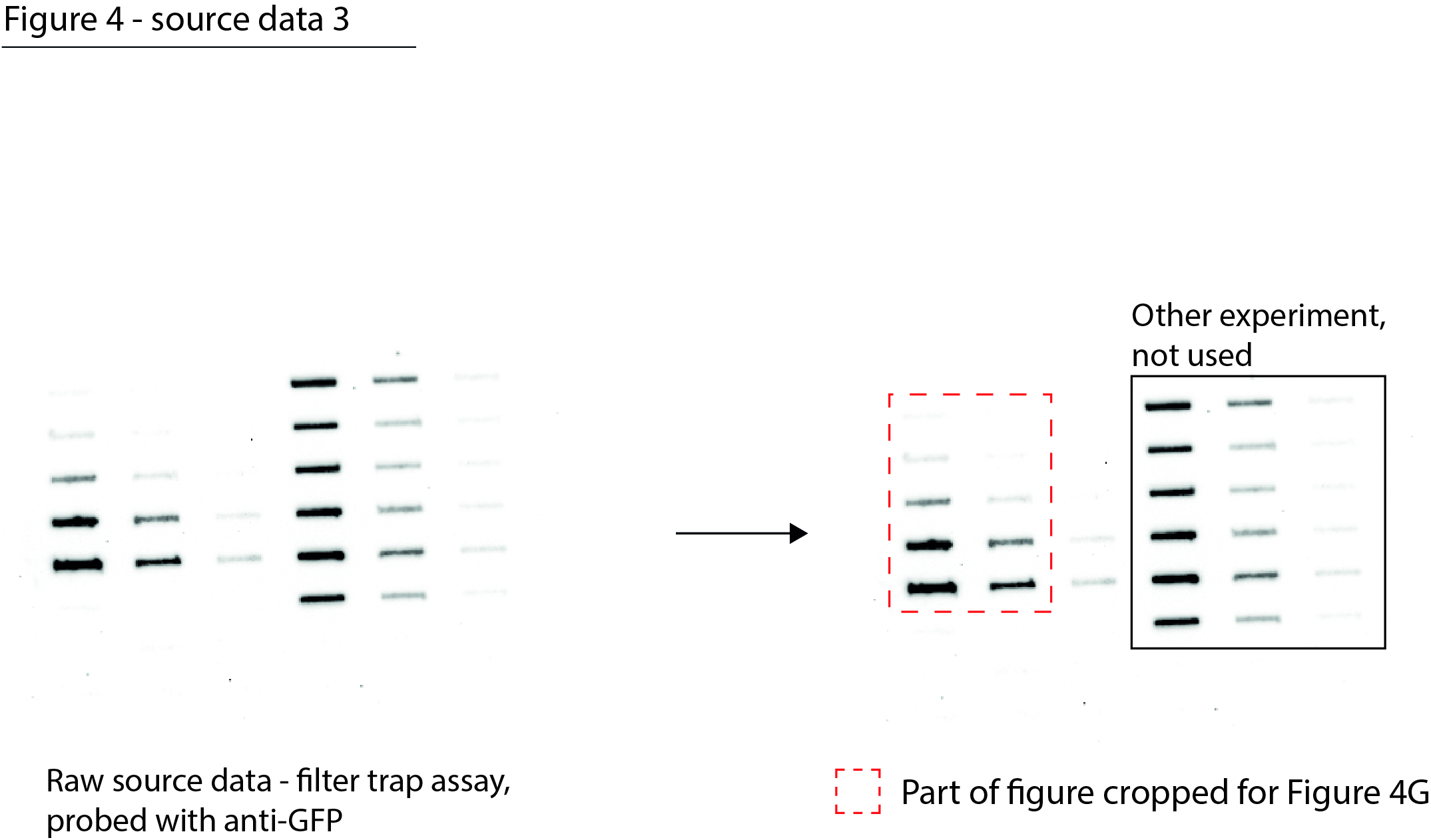

Supplement: Figure 4—source data 3. — Raw filter trap image with the cropped parts indicated in red. [file elife-70726-fig4-data3.jpg]

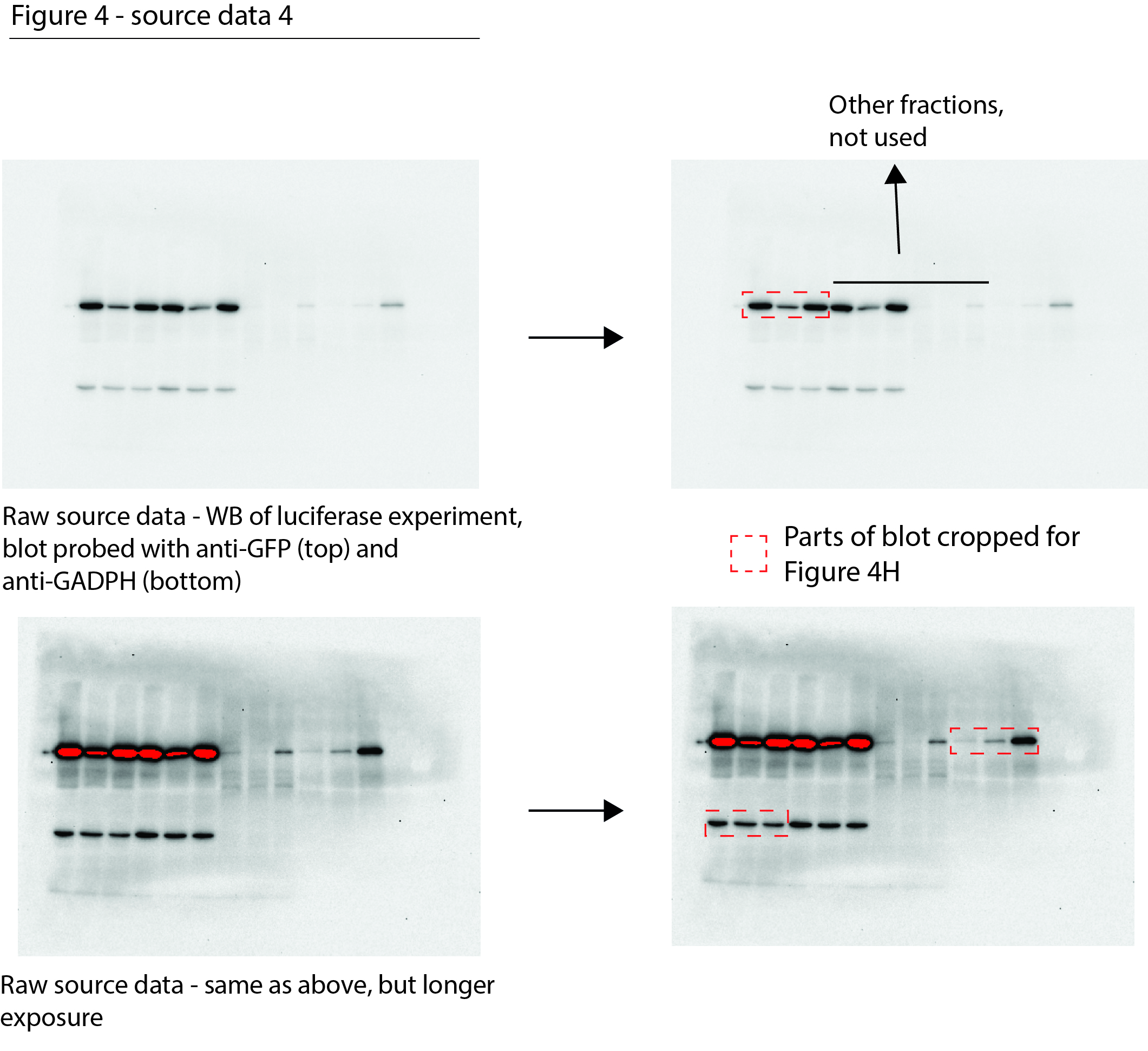

Supplement: Figure 4—source data 4. — Raw Western blot images with each antibody annotated and cropped parts indicated in red. [file elife-70726-fig4-data4.jpg]

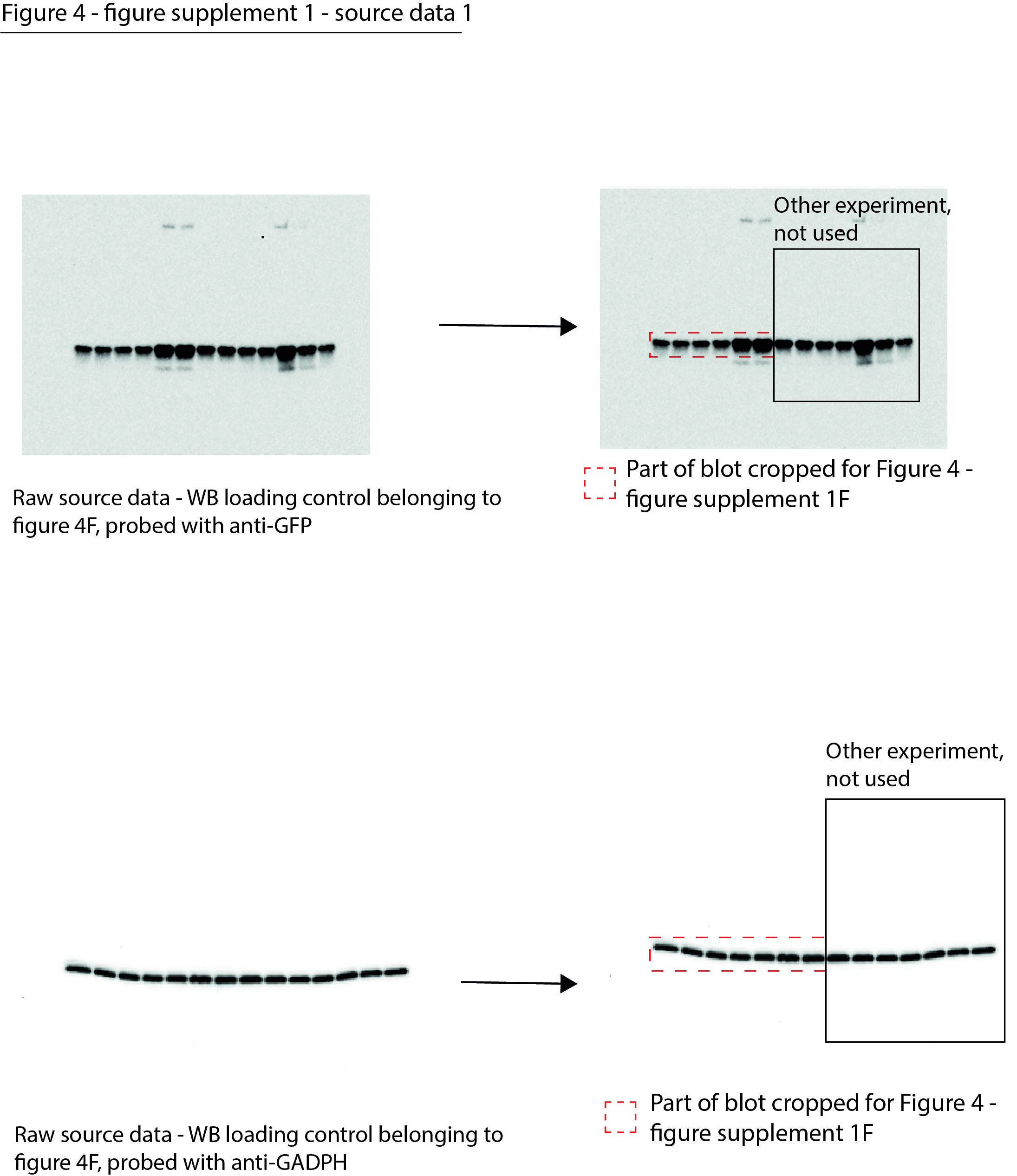

Supplement: Figure 4—figure supplement 1—source data 1. — Raw Western blot images with the antibodies annotated and the cropped parts indicated in red. [file elife-70726-fig4-figsupp1-data1.jpg]

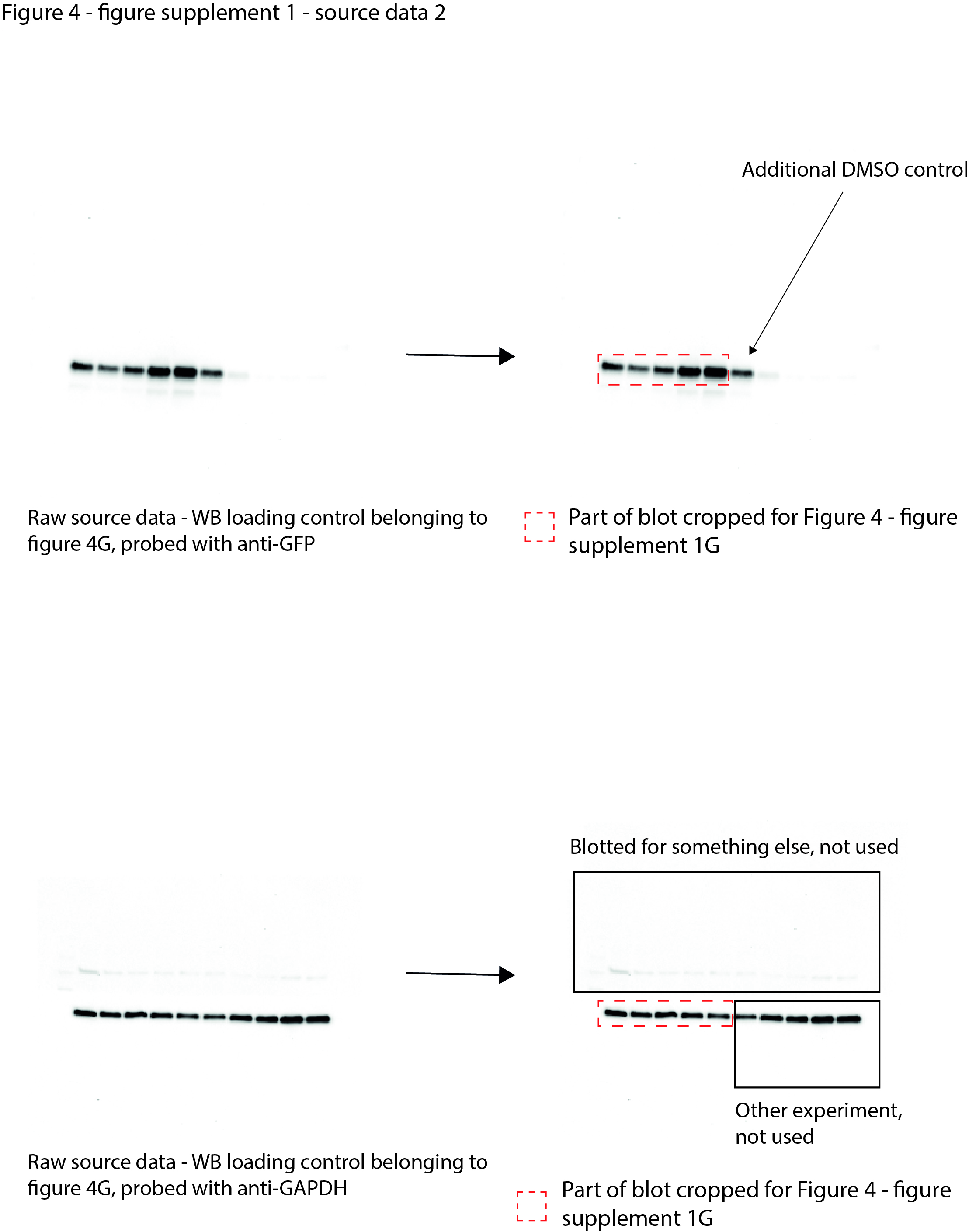

Supplement: Figure 4—figure supplement 1—source data 2. — Raw Western blot images with the antibodies annotated and the cropped parts indicated in red. [file elife-70726-fig4-figsupp1-data2.jpg]

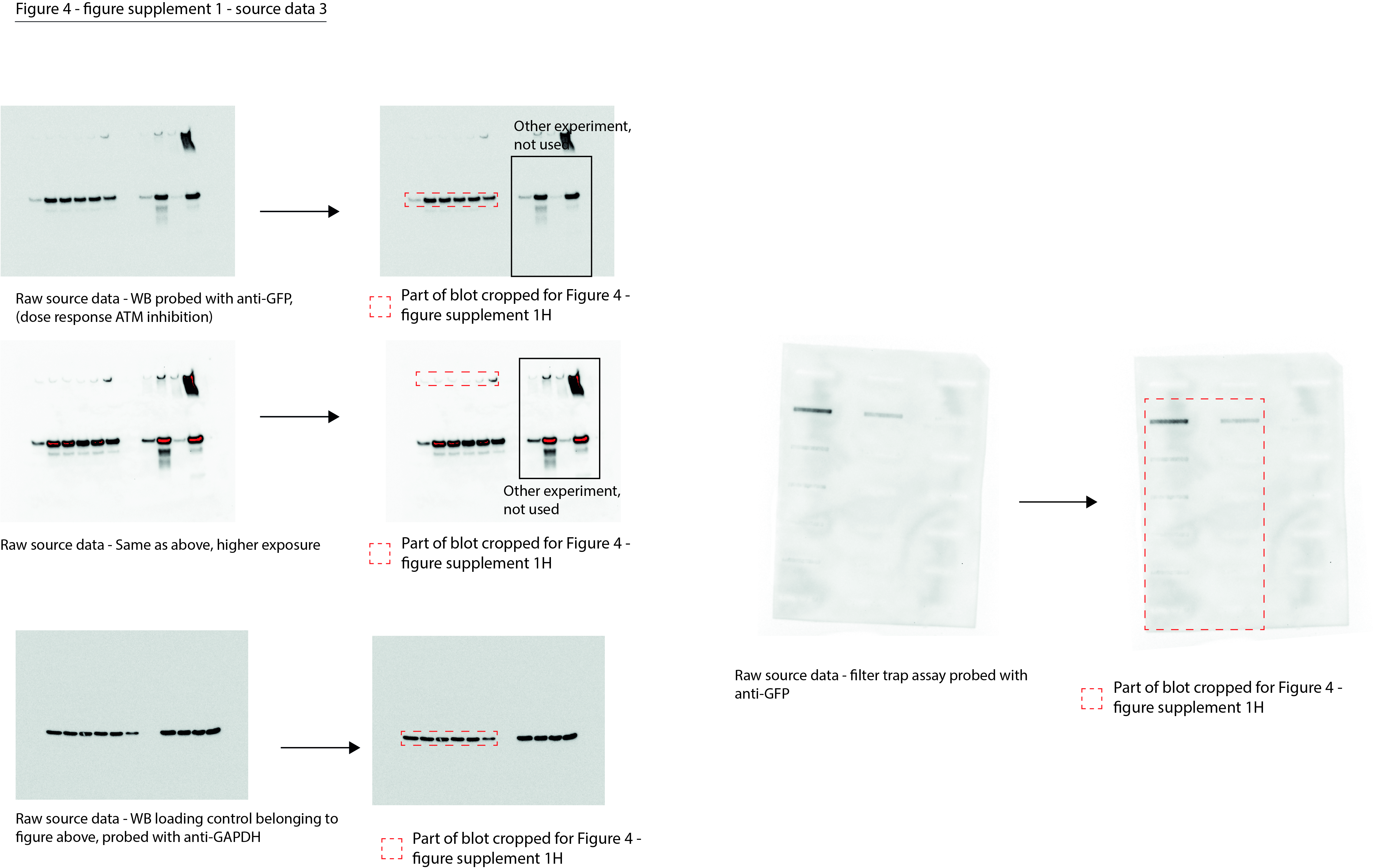

Supplement: Figure 4—figure supplement 1—source data 3. — Raw Western blot images with the antibodies annotated and the cropped parts indicated in red. [file elife-70726-fig4-figsupp1-data3.jpg]

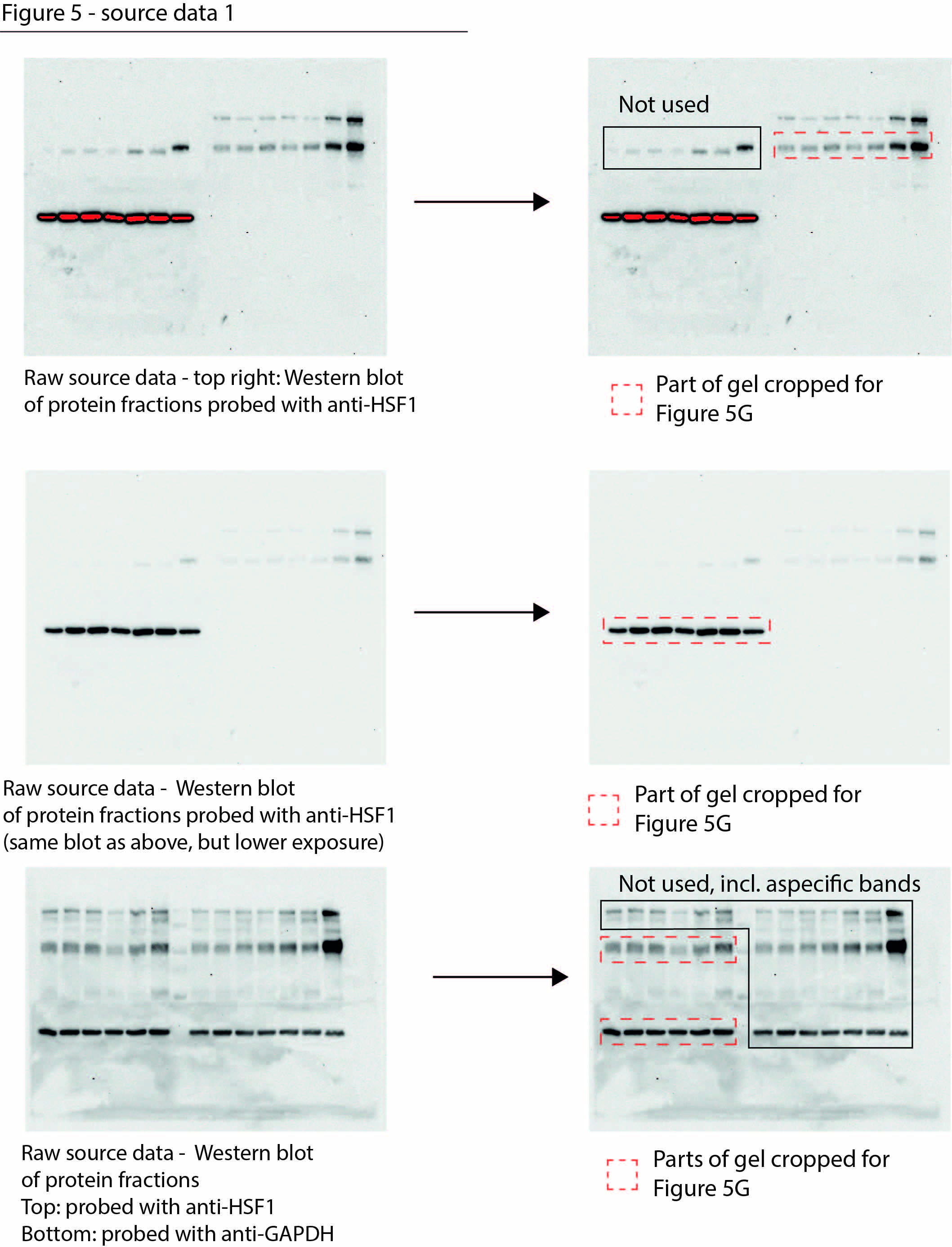

Supplement: Figure 5—source data 1. — Raw Western blot images with each antibody annotated and cropped parts indicated in red. [file elife-70726-fig5-data1.jpg]

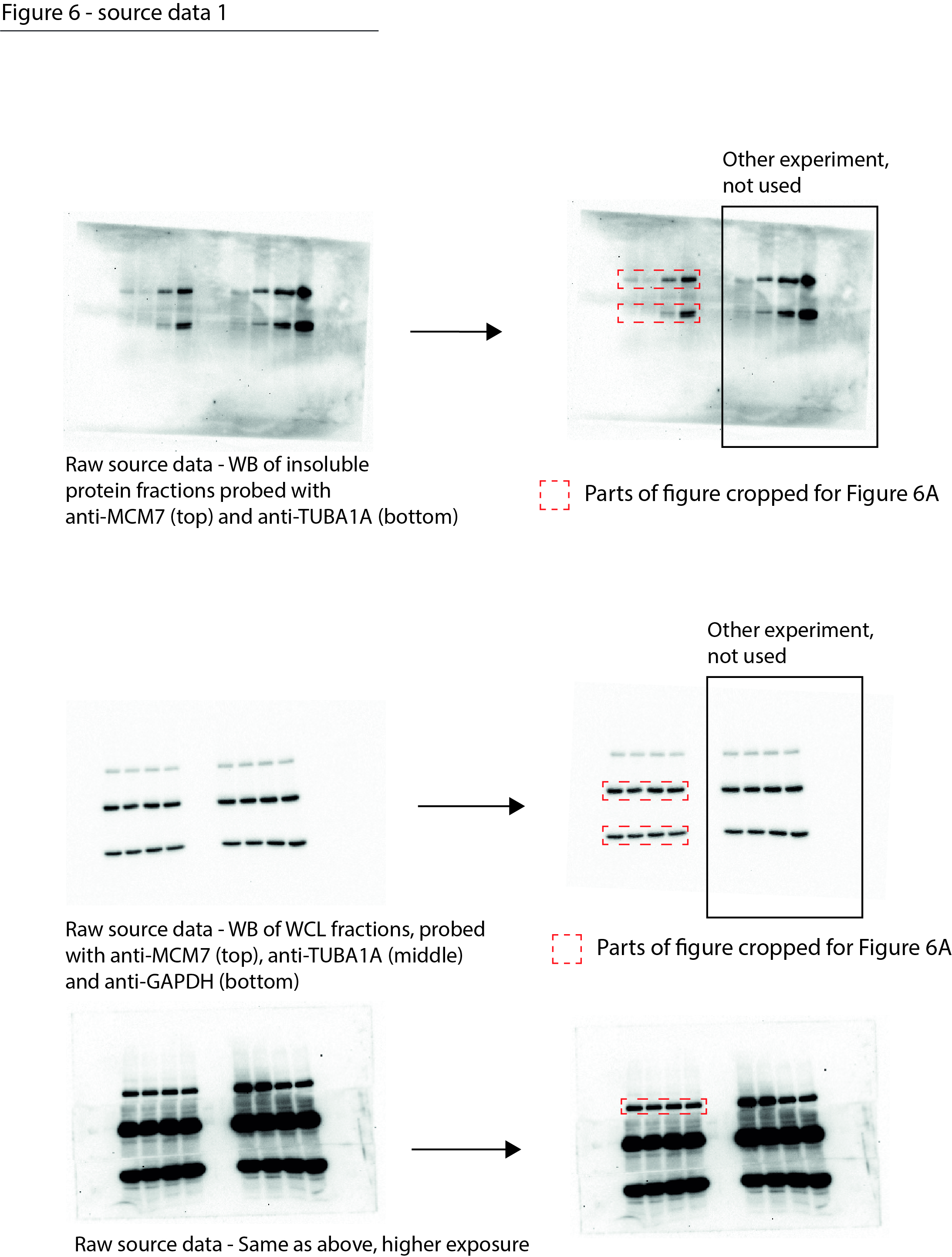

Supplement: Figure 6—source data 1. — Raw Western blot images with each antibody annotated and cropped parts indicated in red. [file elife-70726-fig6-data1.jpg]

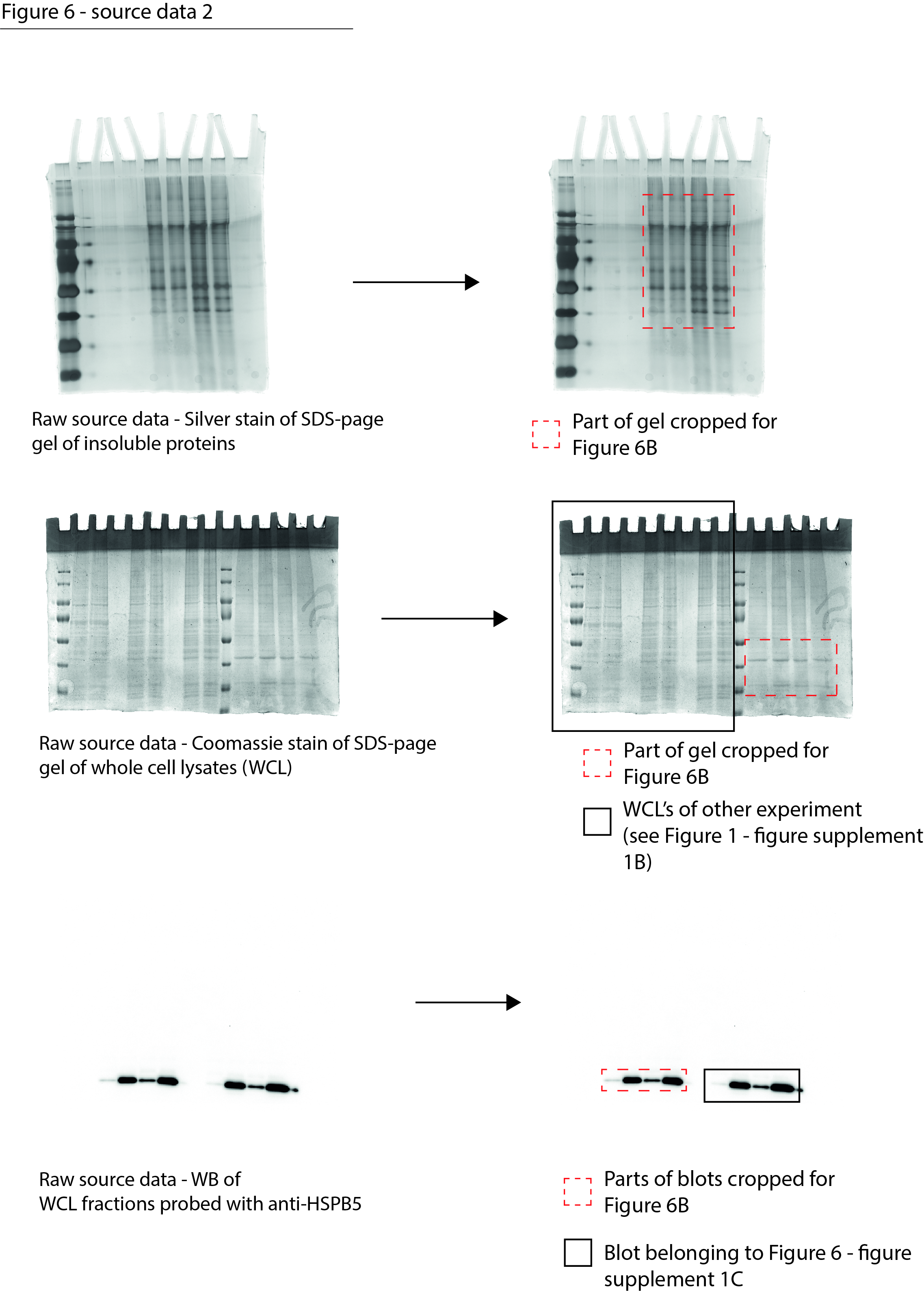

Supplement: Figure 6—source data 2. — Raw Coomassie- and silver-stained SDS-PAGE gels and Western blot images with cropped parts indicated in red. [file elife-70726-fig6-data2.jpg]

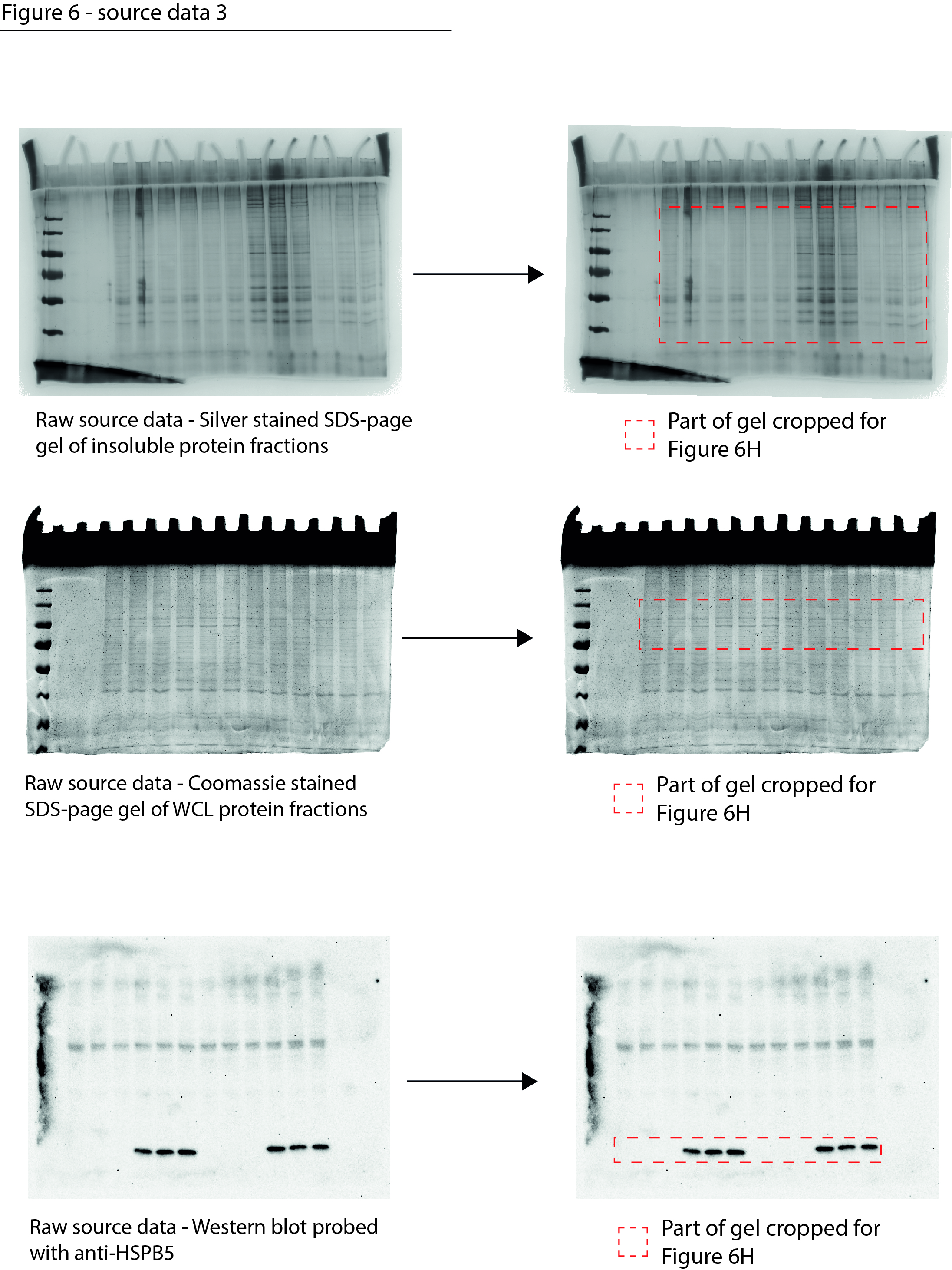

Supplement: Figure 6—source data 3. — Raw Coomassie- and silver-stained SDS-PAGE gels and Western blot images with cropped parts indicated in red. [file elife-70726-fig6-data3.jpg]

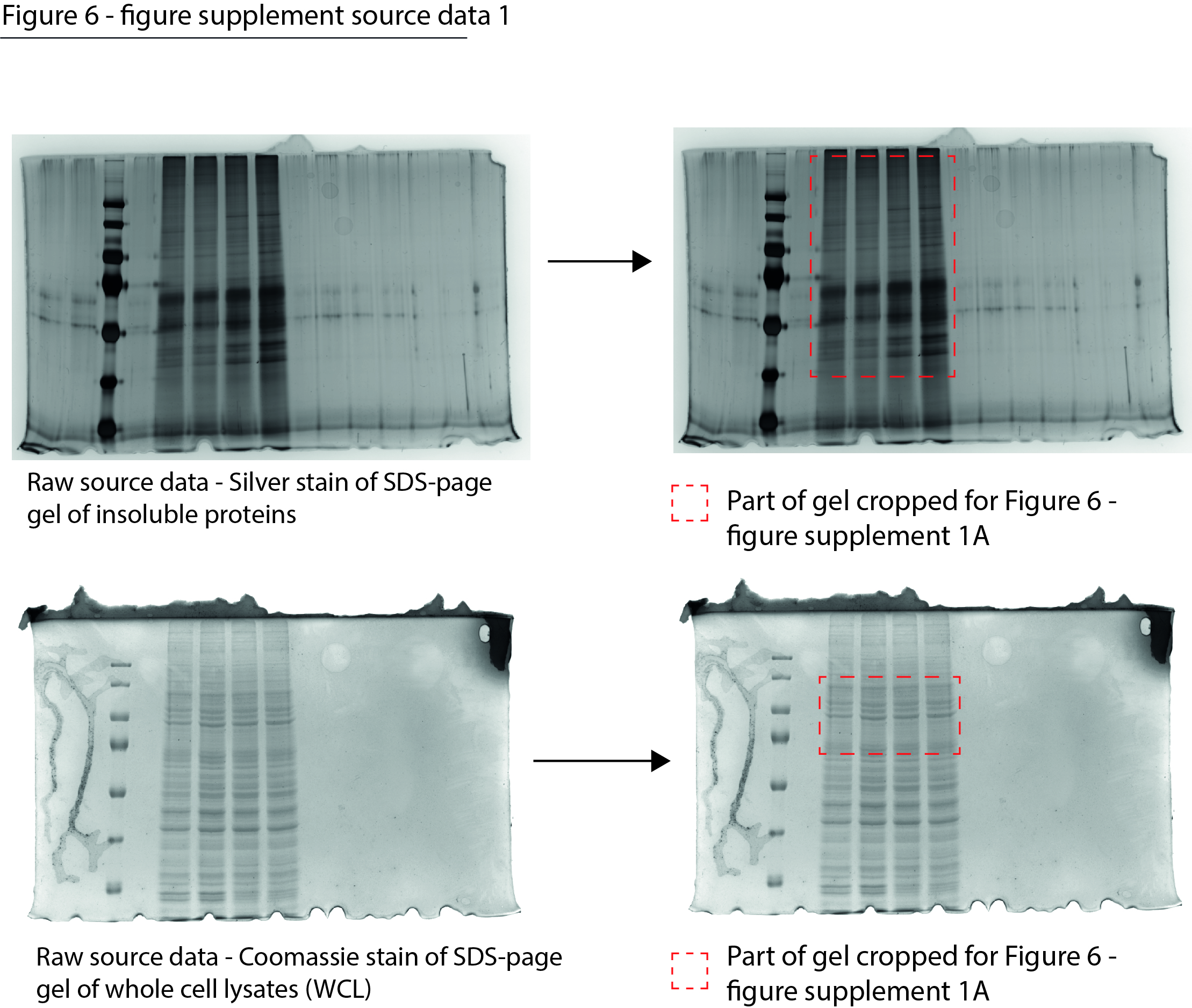

Supplement: Figure 6—figure supplement 1—source data 1. — Raw Coomassie- and silver-stained SDS-PAGE gels with the cropped parts indicated in red. [file elife-70726-fig6-figsupp1-data1.jpg]

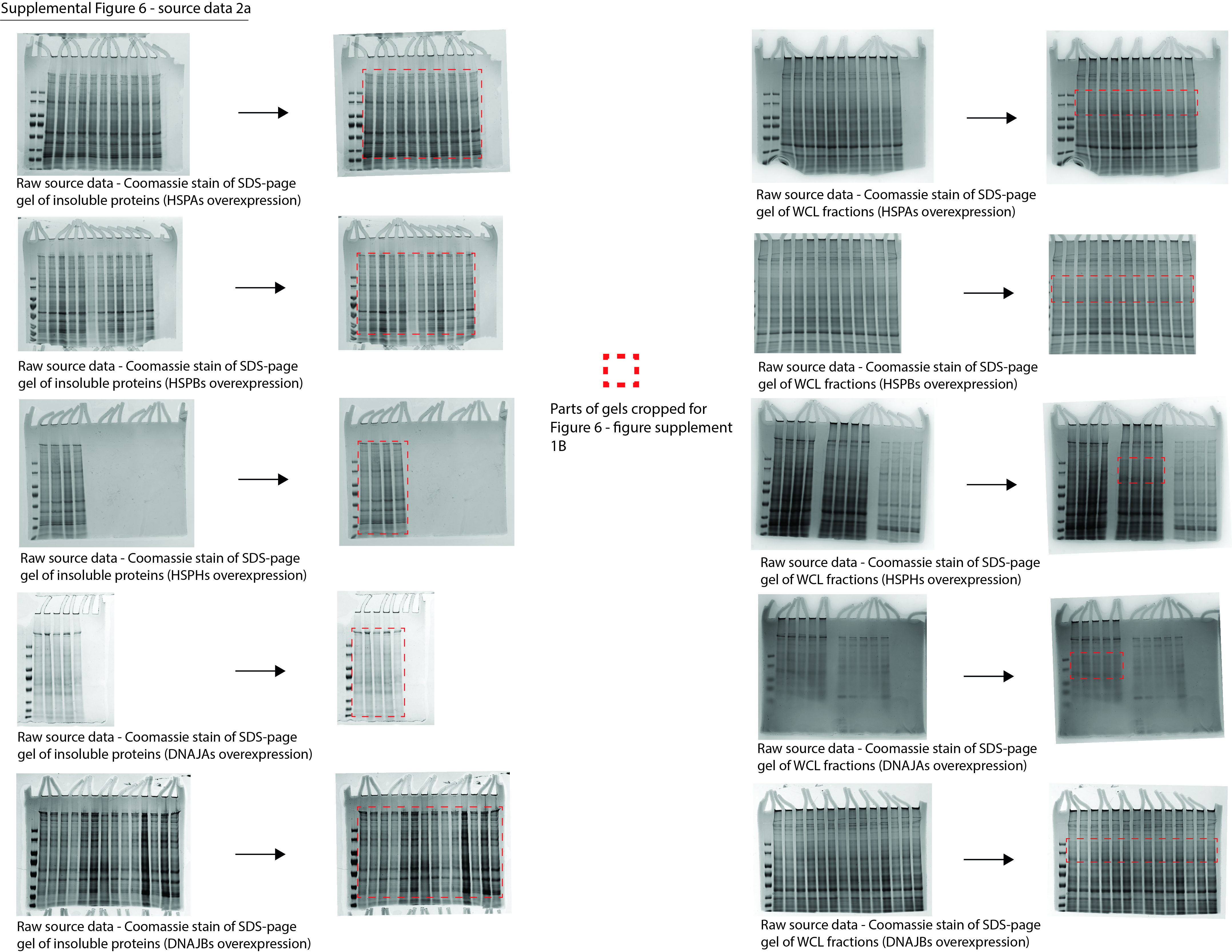

Supplement: Figure 6—figure supplement 1—source data 2. — Raw Coomassie-stained SDS-PAGE gels with the cropped parts indicated in red. Raw Western blot images with the antibodies annotated and the cropped parts indicated in red. [file elife-70726-fig6-figsupp1-data2.zip › Source data - Figure 6 - figure supplement 1 - 2a.jpg]

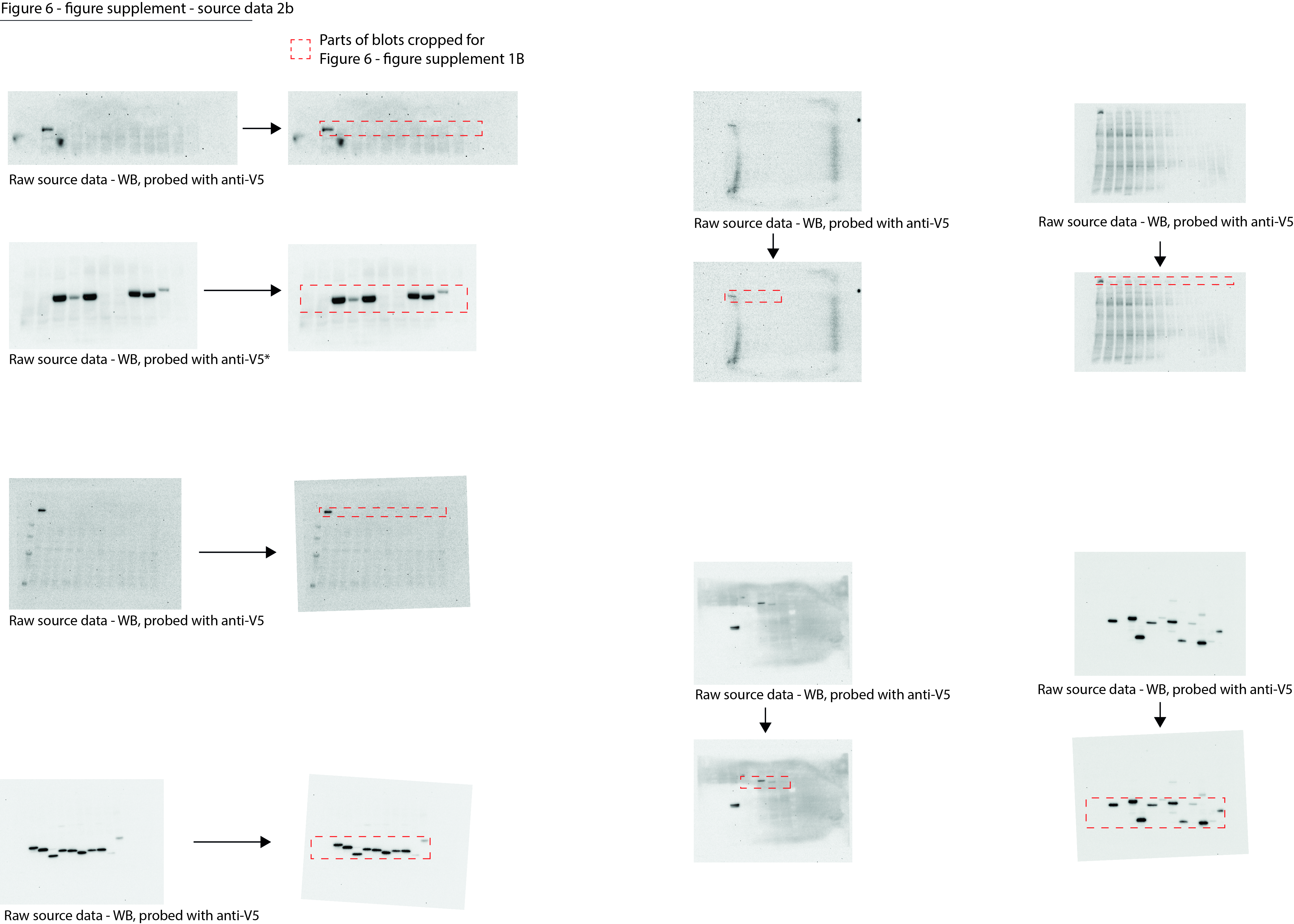

Supplement: Figure 6—figure supplement 1—source data 2. — Raw Coomassie-stained SDS-PAGE gels with the cropped parts indicated in red. Raw Western blot images with the antibodies annotated and the cropped parts indicated in red. [file elife-70726-fig6-figsupp1-data2.zip › Source data - Figure 6 - figure supplement 1 - 2b.jpg]

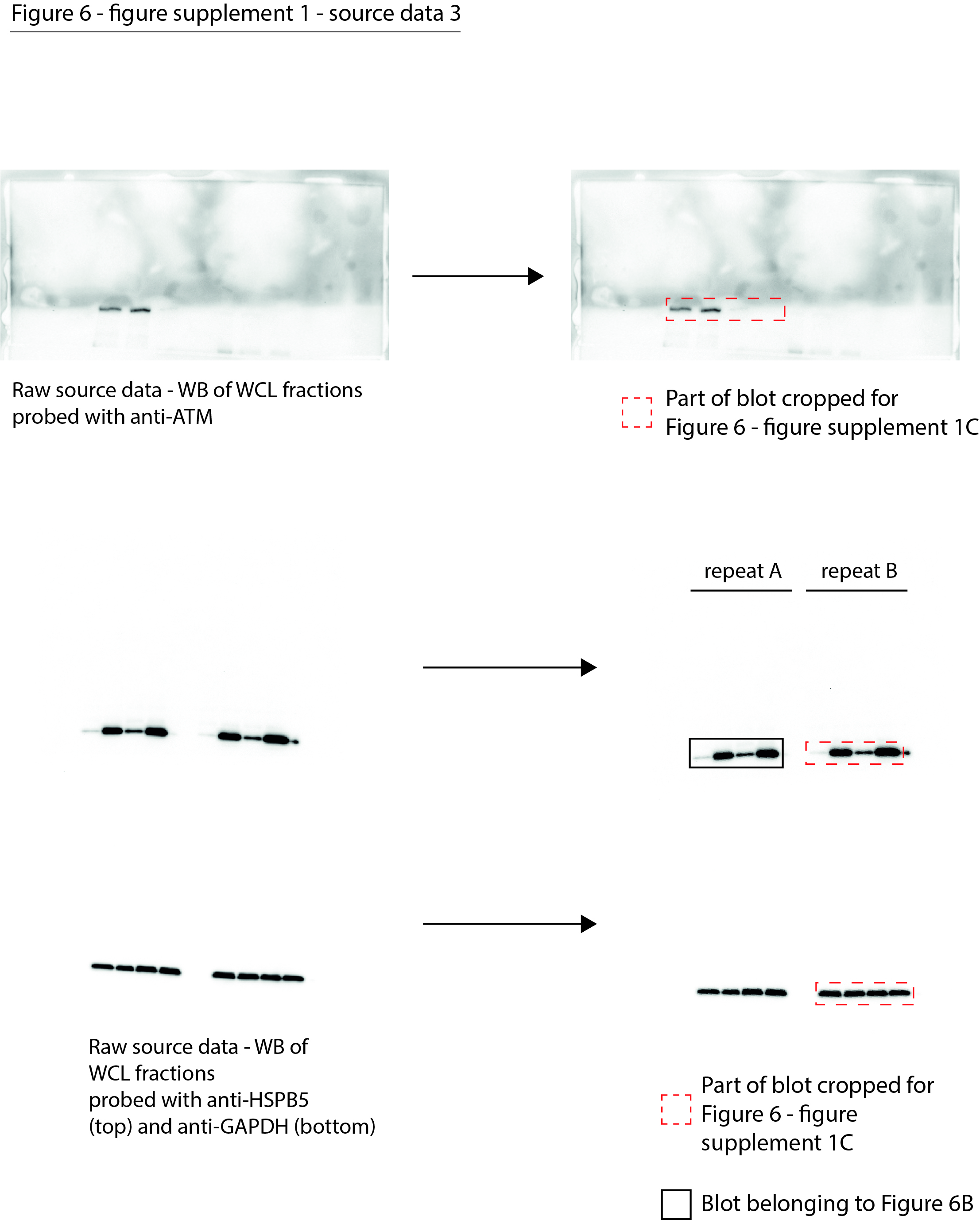

Supplement: Figure 6—figure supplement 1—source data 3. — Raw Western blot images with the antibodies annotated and the cropped parts indicated in red. [file elife-70726-fig6-figsupp1-data3.jpg]

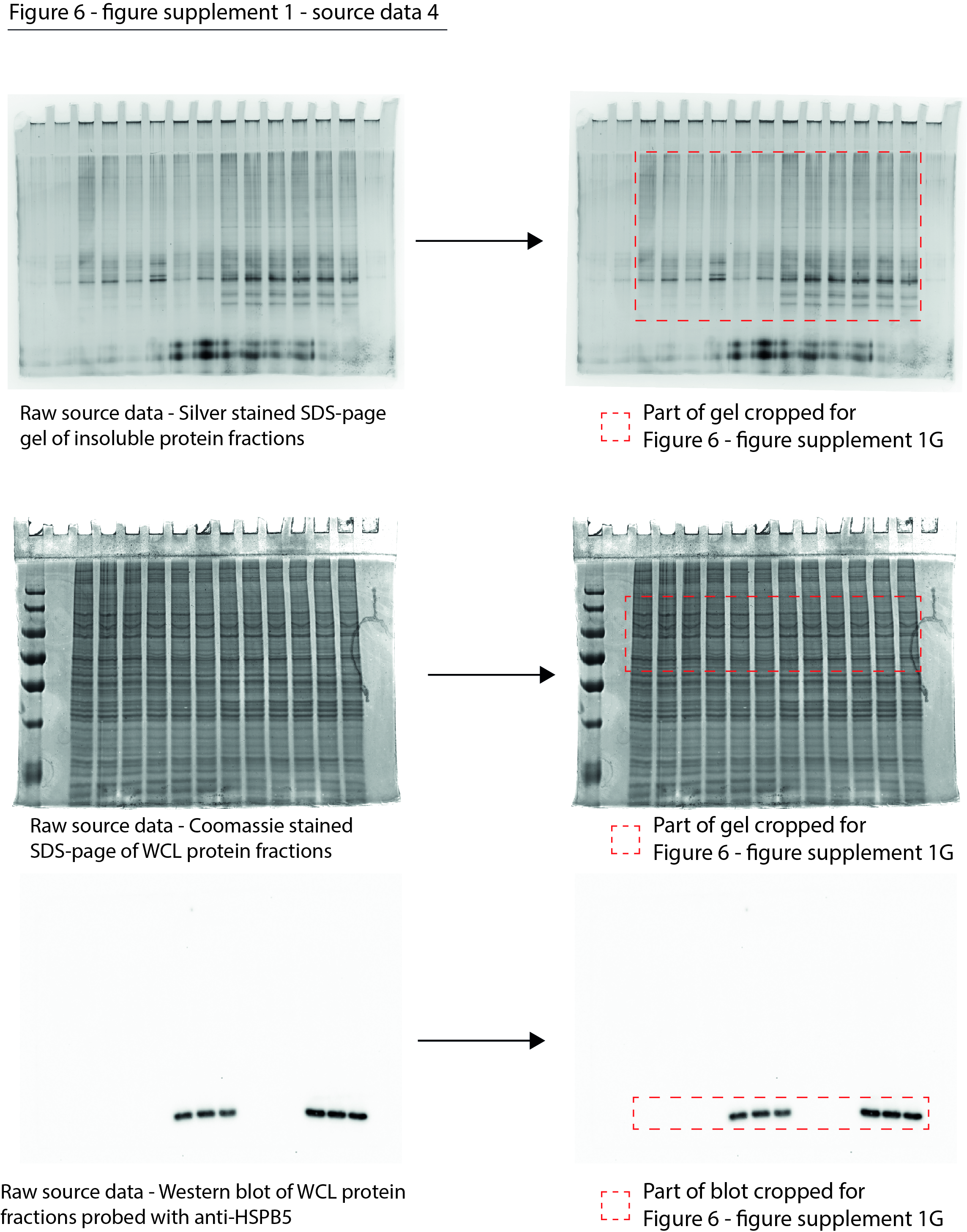

Supplement: Figure 6—figure supplement 1—source data 4. — Raw Coomassie- and silver-stained SDS-PAGE gels and Western blot images with the antibodies annotated and the cropped parts indicated in red. [file elife-70726-fig6-figsupp1-data4.jpg]
