# Supplementary material for: Targeting DNA topoisomerases or checkpoint kinases results in an overload of chaperone systems, triggering aggregation of a metastable subproteome
Source: eLife. 2022 Feb 24;11:e70726. doi: 10.7554/eLife.70726 (PMC8871389; doi:10.7554/eLife.70726)
Supplement: Transparent reporting form [file elife-70726-transrepform1.docx]

***eLife’s* transparent reporting form**

We encourage authors to provide detailed information *within their submission* to facilitate the interpretation and replication of experiments. Authors can upload supporting documentation to indicate the use of appropriate reporting guidelines for health-related research (see [EQUATOR Network](http://www.equator-network.org/%20)), life science research (see the [BioSharing Information Resource](https://biosharing.org/)), or the [ARRIVE guidelines](http://www.plosbiology.org/article/info:doi/10.1371/journal.pbio.1000412) for reporting work involving animal research. Where applicable, authors should refer to any relevant reporting standards documents in this form.

If you have any questions, please consult our Journal Policies and/or contact us: [editorial@elifesciences.org](mailto:editorial@elifesciences.org).

**Sample-size estimation**

- You should state whether an appropriate sample size was computed when the study was being designed
- You should state the statistical method of sample size computation and any required assumptions
- If no explicit power analysis was used, you should describe how you decided what sample (replicate) size (number) to use

Please outline where this information can be found within the submission (e.g., sections or figure legends), or explain why this information doesn’t apply to your submission:

Sample sizes were not computed beforehand. While for some experiments 2 independent biological repeats were performed (indicated in legends when this is the case), all key experiments were performed at least 3 times using independent biological repeats. This is widely accepted in the field. MS/MS and RNAseq were performed 4 times.

**Replicates**

- You should report how often each experiment was performed
- You should include a definition of biological versus technical replication
- The data obtained should be provided and sufficient information should be provided to indicate the number of independent biological and/or technical replicates
- If you encountered any outliers, you should describe how these were handled
- Criteria for exclusion/inclusion of data should be clearly stated
- High-throughput sequence data should be uploaded before submission, with a private link for reviewers provided (these are available from both GEO and ArrayExpress)

Please outline where this information can be found within the submission (e.g., sections or figure legends), or explain why this information doesn’t apply to your submission:

The number of replicates is either indicated in the graphs as individual dots or squares, or can be found in the legends.

Only biological replicates were included in graphs, these originate from independent experiments (i.e. independent cultures/cell lysates etc, different days at which experiments were performed). Where technical repeats (i.e. same cultures/cell lysates, processed together in the same experiment) are shown this is explicitly mentioned.

Data was only excluded if there was a clear indication that the experiment failed due to technical reasons.

All high-throughput and large datasets are available (links are stated in the manuscript).

R-codes used for MS/MS and RNAseq analysis will be made publicly available in the event of acceptance

**Statistical reporting**

- Statistical analysis methods should be described and justified
- Raw data should be presented in figures whenever informative to do so (typically when N per group is less than 10)
- For each experiment, you should identify the statistical tests used, exact values of N, definitions of center, methods of multiple test correction, and dispersion and precision measures (e.g., mean, median, SD, SEM, confidence intervals; and, for the major substantive results, a measure of effect size (e.g., Pearson's r, Cohen's d)
- Report exact p-values wherever possible alongside the summary statistics and 95% confidence intervals. These should be reported for all key questions and not only when the p-value is less than 0.05.

Please outline where this information can be found within the submission (e.g., sections or figure legends), or explain why this information doesn’t apply to your submission:

(For large datasets, or papers with a very large number of statistical tests, you may upload a single table file with tests, Ns, etc., with reference to sections in the manuscript.)

All statistical methods used are stated in the figure legends, and are again outlined in the Materials and Methods section. For each experiment, exact P-values are given as well, and n-values and definitions of center (either mean of median) are shown and stated in the legends. In the few bar graphs in which individual values are not shown, dispersion is shown and indicated in the accompanying figure legend. Raw data is included in the manuscript and publicly available as indicated in the Materials and Methods (for the large RNA sequencing and MS/MS data).

**Group allocation**

- Indicate how samples were allocated into experimental groups (in the case of clinical studies, please specify allocation to treatment method); if randomization was used, please also state if restricted randomization was applied
- Indicate if masking was used during group allocation, data collection and/or data analysis

Please outline where this information can be found within the submission (e.g., sections or figure legends), or explain why this information doesn’t apply to your submission:

Samples were allocated into groups based on their treatment. This is clearly indicated in both the text and the figure legends.

**Additional data files (“source data”)**

- We encourage you to upload relevant additional data files, such as numerical data that are represented as a graph in a figure, or as a summary table
- Where provided, these should be in the most useful format, and they can be uploaded as “Source data” files linked to a main figure or table
- Include model definition files including the full list of parameters used
- Include code used for data analysis (e.g., R, MatLab)
- Avoid stating that data files are “available upon request”

Please indicate the figures or tables for which source data files have been provided:

Source data for all Western blots and gels is uploaded separately (see below).

MS/MS and RNAseq source data files have been uploaded as Supplemental Tables 1 and 2, respectively. They are also available online as indicated in the Materials and Methods. The raw data for these are available as indicated in the Materials and Methods. The R-code used for RNAseq and MS/MS analysis will be made publicly available in the event of acceptance.

GRAPHICAL SOURCE DATA (26 files total) PROVIDED FOR:

Figure 1A, C, E, G;

Figure 1 – figure supplement 1A, B, E, F;

Figure 2D, G

Figure 4D, F, G, H

Figure 4 – figure supplement 1G, H, I

Figure 5G

Figure 6A, B, H

Figure 6 – figure supplement 1A, B, C, G
